# Oxidative Desulfurization Catalyzed by Phosphotungstic Acid Supported on Hierarchical Porous Carbons

**DOI:** 10.3390/nano11092369

**Published:** 2021-09-12

**Authors:** Bao Wang, Lihua Kang, Mingyuan Zhu

**Affiliations:** 1 College of Chemistry and Chemical Engineering, Yantai University, Yantai 264005, China; m13150400939@163.com (B.W.); lhkang@ytu.edu.cn (L.K.); 2 School of Chemistry and Chemical Engineering, Shihezi University, Shihezi 832000, China

**Keywords:** oxidative desulfurization, hierarchical porous carbon, HPW loading, dispersion

## Abstract

A hierarchical porous carbon material (HPC) with an ultra-high specific surface area was synthesized with sisal fiber (SF) as a precursor, and then H_3_PW_12_O_40_·24H_2_O (HPW) was immobilized on the support of SF-HPC by a simple impregnation method. A series characterization technology approved that the obtained SF-HPC had a high surface area of 3152.46 m^2^g^−1^ with micropores and macropores. HPW was well-dispersed on the surface of the SF-HPC support, which reduced the loading of HPW to as low as 5%. HPW/SF-HPW showed excellent catalytic performance for oxidative desulfurization, and the desulfurization rate reached almost 100% under the optimal reaction conditions. The desulfurization rate of HPW/SF-HPW could be maintained at above 94% after four recycles.

## 1. Introduction

Reduction in the sulfur content in oil products is the primary solution to decrease the pollution caused by oil burning [1]. Among the developed desulfurization technologies, oxidative desulfurization is widely studied as the most promising process because of its mild reaction conditions and good desulfurization effect [2]. In this approach, heteropoly acids such as phosphotungstate decompose in the presence of excessive hydrogen peroxide into a peroxide metal complex W(O_2_)_n_, providing an active site for oxidative desulfurization [3,4]. For catalyst separation and recycling, it is desirable to load the heteropoly acids onto support materials [5].

In this context, Yang et al. [6] prepared a m/M-HPW/SiO_2_-20 catalyst by loading 12-tungstophosohoric acid (HPW) on multi-stage porous silica. Under optimal conditions, the removal rate of dibenzothiophene (DBT) was found to reach 100%. However, the specific surface area of the m/M-HPW/SiO_2_-20 catalyst was only 346 m^2^g^−1^, and the aperture was less than 10 nm, making the HPW loading as high as 20%. Meanwhile, Yue et al. [7] studied the loading of HPW on hierarchical ordered silica with the aim of adjusting the pore structure of the catalyst, which not only maintained a high specific surface area but also provided structures with different pore sizes and improved the performance of the catalyst. Under optimum conditions, 95.1% sulfur was removed after eight cycles. Huang et al. [8] used sodium-dodecyl-benzene-sulfonate-modified layered double hydroxide as a support to load HPW for oxidative desulfurization. Under optimal conditions, the sulfur removal rate was close to 100%, and after 15 cycles the removal rate was reduced to 95.73%. Although the specific surface area was only 167 m^2^g^−1^, the pore diameter of the catalyst was 12.99 nm, and this large pore size increased the reaction mass transfer rate, thus allowing the HPW load to be reduced to 10%. Pham et al. [9] prepared a PW-NH^3+^-SBA-15 catalyst. Under the optimal conditions, the conversion rates of DBT and BT reached 100% and 99.9%, respectively. The catalyst can be reused four times without a significant decrease in catalytic activity. The change in catalyst structure confirmed a strong interaction between the SBA-15 support and the HPW catalytically active site. Gao et al. [10] incorporated HPW into TiO_2_ pellets to improve catalytic activity and recyclability. After seven runs, it had good conversion and selectivity. However, these excellent catalysts often required high loading [11,12,13]. The content of HPW will affect the quality of the oil, and the phosphorus will cause environmental problems. Therefore, it is very important to reduce the load of HPW. The large content of HPW required for the reaction is mainly due to the fact that HPW is prone to agglomeration [14]. Therefore, to reduce the loading of HPW, it is necessary to prevent agglomeration and increase its dispersion on the surface of the support, thereby providing sufficient active sites. To ensure a high dispersibility, the support must have a high specific surface area. Although the presence of micropores can increase the specific surface area, it also weakens the mass transfer effect, which is disadvantageous for the reaction of oxidative desulfurization. Therefore, to reduce the HPW load on the support, a material with a large surface area and pore structure is desirable.

Hierarchical porous carbons (HPCs) have not only high specific surface areas but also large pore structures [15]. In many applications, specific surface area is the most important structural parameter. Li et al. [16] adjusted the porosity of carbon materials by controlling the template removal strategy. The method simplified the preparation process and produced honeycomb carbon with a macroporous/mesoporous/microporous-scaled pore structure with a specific surface area of up to 1011 m^2^g^−1^. Supercapacitors assembled with porous carbon as electrodes exhibited large specific capacitances and provided good cycle stability. Liu et al. [17] combined the electrospinning technology, the in situ polymerization and carbonization processes, and the manufacturing process to produce a nitrogen-doped graded porous carbon fiber material, which can remove organic dyes efficiently. It also mentioned that in multi-stage pore materials, macropores can provide efficient mass transfer [18,19], while micropores/mesopores provided a large surface area for the dispersion of HPW [20,21,22].

In this study, we describe the use of a HPC as a support for a HPW catalyst. The high specific surface area of HPC improved the dispersion of HPW and avoided its agglomeration, while the macroporous structure increased the reaction rate and thus allowed the HPW loading to be reduced. In addition, the catalytic performance of the catalyst in the oxidative desulfurization reaction was investigated, the reaction conditions were optimized, and the stability of the catalyst was explored.

## 2. Materials and Methods

### 2.1. Materials

Sisal fiber was procured from Yiwu Shepai Crafts Firm (Yiwu, Zhejiang, China). KOH was purchased from Tianjin Shengao Chemical Reagent Co., Ltd. (Tianjin, China). Hydrochloric acid was was procured by Nanjing Chemical Reagent Co., Ltd. (Nanjing, Jiangsu, China) HPW (AR) was produced by ChengDu KeLong Chemical Co., Ltd. (Chengdu, Sichuan, China). N-Octane was supplied from Sinopharm Chemical Reagent Co., Ltd. (Shanghai, China). DBT (99%) was procured from J&K Scientific Company (Beijing, China). Benzothiophene (BT, 97%), thiophene(Th, 99%), and 4, 6-dimethyldibenzothiophene (4, 6-DMDBT, 99%) were procured from Aladdin Biochemical Technology Co., Ltd. (Shanghai, China). Hydrogen peroxide was purchased from Tianjin Yongsheng Fine Chemical Co., Ltd. (Tianjin, China). Acetone and methanol were produced by Tianjin FuYu Chemical Co., Ltd. (Tianjin, China). All reagents were used without any purification.

### 2.2. Synthesis of HPW/SF-HPC

The hierarchical porous materials were prepared following procedures found in the literature [19]. The sisal fiber was cut into small pieces before use and pre-carbonized at 550 °C for 3 h in a N_2_ atmosphere. The obtained sample was labeled as SF. SF and KOH were then mixed at a mass ratio of 1:5, adding deionized water to dissolve the KOH pellets. After mixing evenly, the mixture was allowed to stand for 1 h to ensure complete immersion of SF in the KOH solution. Then, the mixture was dried for 6 h at 100 °C, and carbonized at 900 °C for 3 h in a N_2_ atmosphere. The mixture sample was washed with dilute hydrochloric acid and deionized water to remove unreacted KOH. The wet sample was dried in an oven at 80 °C overnight, and the solid powder was collected and labeled as SF-HPC.

The catalysts were prepared as follows: after dissolving 0.05 g of HPW in 10 mL of deionized water, 0.95 g of SF-HPC was added and stirred at room temperature for 24 h. A solid sample, which was marked as 5%HPW/SF-HPC, was obtained. The sample was collected by filtration and dried at 80 °C. In a similar manner, the catalysts 1%, 10%, and 20%HPW/SF-HPC were prepared by varying the amount of HPW.

### 2.3. Characterization

Transmission electron microscopy (TEM) experiments were conducted on a Tecnai G2 F20 S-TWIN (Hillsboro, OR, USA) (200 kV) instrument. The structural parameters of the material were obtained from the Micromeritics Model ASAP 2460 (Atlanta, GA, USA) instrument by nitrogen absorption and desorption experiment at 77 K. Powder X-ray diffraction (XRD) data were collected on a Bruker (Karlsruhe, Baden-Württemberg, DEU) advanced D8 X-ray diffractometer operating at 40 mA and 40 kV with Cu-Ka irradiation (λ = 0.15406 nm). The surface morphology and structure of the materials were observed by scanning electron microscopy on a Zeiss Sigma 300 (Jena, Freistaat Thüringen, Germany). The Fourier-transform infrared (FTIR) spectroscopy was performed on a Thermo Fisher Nicolet iS10 spectrometer (Waltham, MA, USA). Inductively coupled plasma (ICP) experiments were performed on an Agilent ICP-OES 730 series ICP (Santa Clara, CA, USA). The macroporous structure of the samples was obtained by using a Micromeritics AutoPore IV 9510 mercury porosimeter (Atlanta, GA, USA).

### 2.4. Catalytic Performance Testing

DBT as a sulfur source was dissolved in n-octane to prepare simulated oil with a sulfur content of 100 ppmw, and the desulfurization performance of the catalyst was tested using the resulting solution as follows: 10 mL of simulated oil was added to a 100 mL three-necked flask, and a quantitative amount of catalyst was added and dispersed in the simulated oil by ultrasonication. The three-necked flask was placed in a constant-temperature water bath, and a certain amount of 30% hydrogen peroxide solution was added according to the desired O/S; stirring was started and timing was started. When the desired reaction time was reached, the three-necked flask was taken out of the bath, and the catalyst was allowed to sink into the bottom of the flask. The reaction solution was poured into a 25 mL beaker, and 10 mL of methanol was added for extraction. After the extraction, a part of the supernatant was taken out to test sulfur content with a Coulomb analyzer (WK-2D).

The catalytic performance was evaluated according to the following equation:

$$\mathrm{Sulfur}\;\mathrm{removal}\;\left(\%\right)=\frac{C_{0}-C_{t}}{C_{0}}\times 100\%$$

where $C_{0}$ represents the sulfur concentration in the configured simulated oil and $C_{t}$ refers to the sulfur content in the oil after t, time, measured by the Coulomb analyzer.

## 3. Results and Discussion

### 3.1. Catalyst Characterization

**Figure 1:**
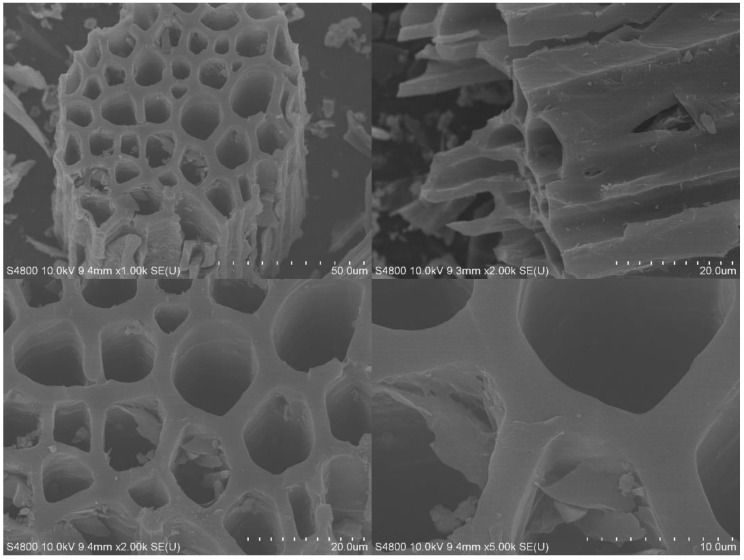
SEM images of SF-HPC.

The scanning electron microscopy images depict the morphology of the SF-HPC sample in Figure 1. As can be seen, the SF-HPC materials had obvious macroporous structures, which provided the basis for preparing the support with a high specific surface area. The average thickness of the honeycomb carbon wall was about 3 μm, which enabled the formation of small holes in the walls. During the KOH treatment, the carbon wall portion was punctured, and the adjacent channel-like macropores communicated with each other. Nevertheless, the intermittent fracture of these carbon substrates did not disrupt the continuity of the macroporous skeleton. In fact, the interconnectivity of the macroporous skeleton was known to improve the mass transfer performance of the material [23,24].

**Figure 2:**
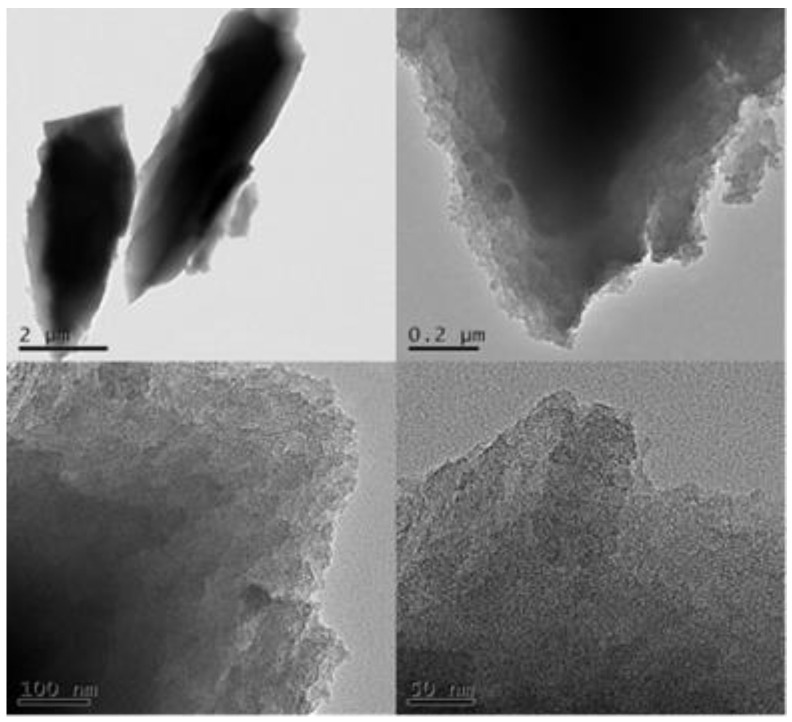
TEM images of SF-HPC.

Figure 2 displays a TEM micrograph of SF-HPC. The magnified image clearly shows that the activated SF-HPC generated a large number of smaller pores on the walls of the macropores [15], which was the main reason for the increase in specific surface area. This was consistent with the results of the nitrogen adsorption and desorption test discussed below.

**Figure 3:**
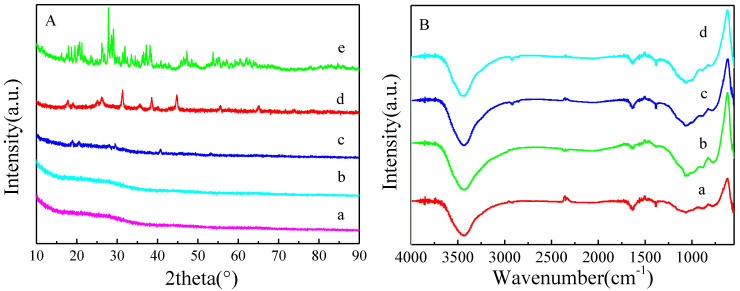
(**A**) XRD patterns of (a) SF-HPC, (b) 1%HPW/SF-HPC, (c) 5%HPW/SF-HPC, (d) 10%HPW/SF-HPC, and (e) HPW. (**B**) FT-IR spectra of (a) SF-HPC, (b) 1%HPW/SF-HPC, (c) 5%HPW/SF-HPC, and (d) 10%HPW/SF-HPC.

The XRD pattern of the support and the catalyst are shown in Figure 3A. It can be seen that SF-HPC had a distinct diffraction peak at 20–30°, corresponding to amorphous carbon. The pattern of 1%HPW/SF-HPC was the same as that of the support SF-HPC, because the low content of HPW was uniformly distributed on the SF-HPC surface, which could not be detected by XRD. In contrast, the characteristic peaks of HPW appear in the pattern of 5% and 10%HPW/SF-HPC, being more obvious in the latter catalyst due to the increased HPW content.

Figure 3B shows the infrared spectra of SF-HPC and the HPW/SF-HPC catalysts. SF-HPC gave rise to absorption peaks at 784.41, 808.87, 872.32, 1383.88, and 2347.29 cm^−1^. Meanwhile, the absorption peak of HPW became more obvious with catalyst loading increasing. In the spectrum of the 10%HPW/SF-HPC catalyst, the peaks that appeared at 795.43, 892.18, 989.50, and 1066.52 cm^−1^ could be attributed to the vibration of the W–Oc–W, W–Ob–W, W–Od, and P–Oa bonds, respectively. Compared to the corresponding characteristic absorption peaks in pure HPW (799.58, 892.26, 982.44, and 1080.24 cm^−1^) [25,26,27], the tensile vibrations of W–Oc–W, W–Ob–W and P–Oa were blue-shifted and that of W–Od was red-shifted, which confirmed the interaction between HPW and SF-HPC.

**Figure 4:**
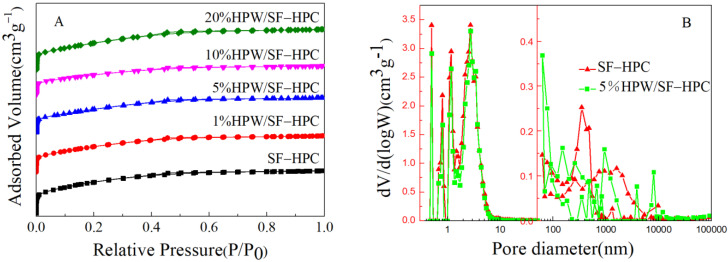
(**A**) Nitrogen adsorption/desorption isotherms of samples. (**B**) Pore size distribution curves of SF-HPC and 5%HPW/SF-HPC.

In order to further understand the structural properties of the catalyst and the support, the support and the catalyst were analyzed by nitrogen adsorption and desorption analysis to obtain physical properties, such as specific surface area, pore volume, and pore size, as shown in Table 1. The specific surface area of SF-HPC in Table 1 was as high as 3152 m^2^g^−1^, which was larger than the specific surface area of HPC reported in the previous literature [20,21]. As shown in Figure 4A, in the N_2_ adsorption isotherm, all isotherms were close to type I, indicating the presence of micropores. In addition, there was a very small hysteresis loop in the range of P/P_0_ in the range of 0.45–0.5, which indicated the presence of a mesoporous structure in the pore structure. It could also be seen from the pore size distribution in Figure 4B. The macroporous structure of the sample was characterized by a mercury intrusion meter. The results showed that there were macroporous structures in the SF-HPC sample. These were consistent with those observed by SEM, which further confirmed that the samples were hierarchical porous materials. After loading HPW, the specific surface area decreased and the pore diameter did not obviously change. It could also be seen from the pore size distribution that the pore size distribution did not greatly change after the loading of HPW.

**Figure 5:**
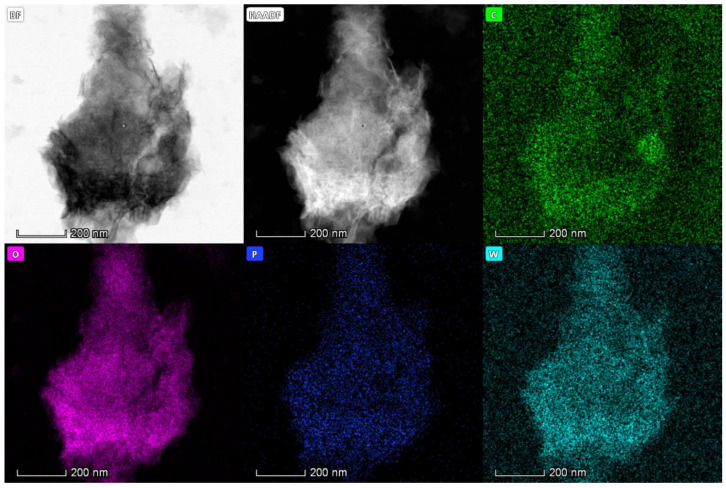
EDS elemental mapping images of 5%HPW/SF-HPC.

To clearly observe the distribution of the active component, HPW, on the surface of the support SF-HPC, we performed an EDS mapping test with the 5%HPW/SF-HPC catalyst. As shown in Figure 5, we could clearly observe phosphorus and tungsten, indicating that HPW had good dispersibility on the surface of the support SF-HPC. This result confirmed the uniform distribution of HPW on the support SF-HPC and the lack of HPW agglomeration, which was consistent with the XRD results.

### 3.2. Catalytic Performance

**Figure 6:**
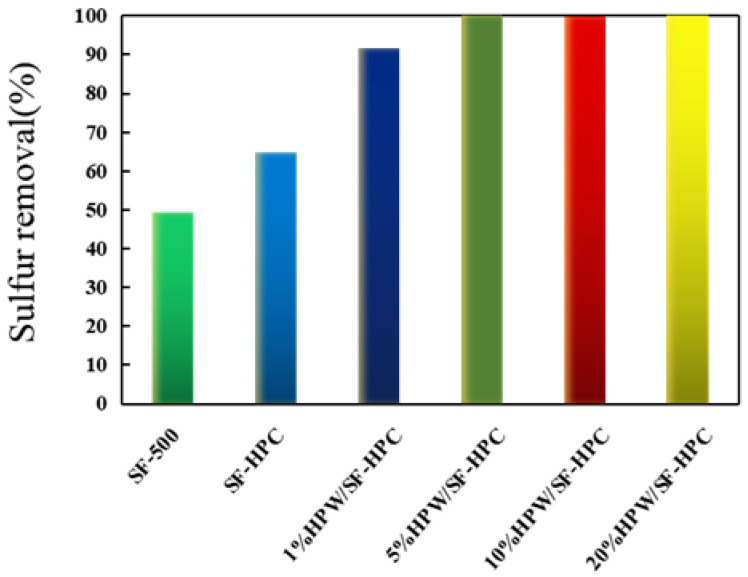
The sulfur removal rate in the ODS process with different HPW contents.

The desulfurization activity of the catalysts was evaluated following the procedure described in the experimental section. Firstly, we investigated the desulfurization effect before and after loading the active component. As shown in Figure 6, the desulfurization effect increased significantly after loading HPW, demonstrating that the active components promoted the desulfurization effect. Furthermore, the desulfurization activity increased with the increase in the loading (Figure 6), reaching 100% with catalyst loading of 5%. The other catalysts containing 10% and 20% loading could also achieve a desulfurization rate of 100%. Consistent with our assumptions, high dispersion of HPW allowed the HPW load to be reduced. Therefore, 5% could be envisaged as the optimal loading, and subsequent experiments were carried out with the 5%HPW/SF-HPC catalyst. Compared to previous reports [11,28,29], a great reduction in the catalyst loading was achieved by using the present HPC support. The HPW content was thereby reduced, which was beneficial for decreasing the effect of phosphorus pollution on the environment.

**Figure 7:**
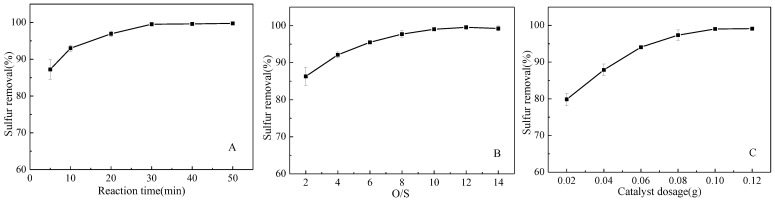
(**A**) Effect of reaction time (T = 25 °C, O/S = 10, and catalyst dosage = 0.1 g/10 mL). (**B**) Effect of O/S molar ratio (T = 25 °C, t = 30 min, and catalyst dosage = 0.1 g/10 mL). (**C**) Effect of catalyst dosage (t = 30 min, T = 25 °C, and O/S = 10).

Other beneficial features of the present process were the reaction temperature, since 100% desulfurization could be achieved at room temperature, and the lack of heat consumption, which would save production costs. Other parameters displayed the relationship between the desulfurization rate and the reaction time as shown in Figure 7A. As can be seen, the desulfurization rate increased with time, reaching 100% at 30 min of reaction time. This constituted a considerable shortening of the previously reported desulfurization time, which was beneficial for industrial applications. Figure 7B shows that the sulfur removal rate improves with the increase in the O/S ratio, reaching 100% for O/S = 10. In contrast, when the O/S ratio reached 14, the desulfurization rate decreased slightly, which may be due to the excessive hydrogen peroxide adsorbing water molecules on some active sites on the catalyst surface, thus reducing the adsorption of DBT [8,30]. A significant effect of the amount of catalyst on the desulfurization effect can be seen in Figure 7C. As the amount of catalyst increased, the sulfur removal rate increased considerably, reaching 100% when the amount of catalyst was 0.1 g. In summary, the optimal parameters of the reaction, which were significantly enhanced compared to previous reports [31,32,33,34], were room temperature, 30 min of reaction time, O/S = 10, and 0.1 g of catalyst per 10 mL of oil.

**Figure 8:**
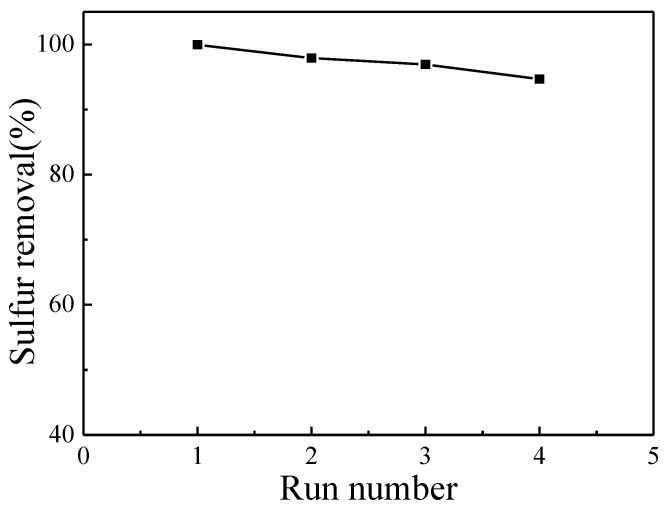
Effect of recycling times on DBT conversion.

To further investigate the performance of the catalyst, we recovered the catalyst and investigated its stability. After the catalytic oxidation reaction was completed, the catalyst was filtered and dried. The dried solid product was collected for the next catalytic reaction. According to the results shown in Figure 8, the desulfurization rate was still maintained at above 94% after four recycles. The loss of HPW was detected by an ICP test. The result is shown in Table 2. The actual load of fresh 5%HPW/SF-HPC catalyst was 4.252% lower than the theoretical value of 5%. This was due to the fact that some HPW remained in the solution during the loading process. As the number of cycles increased, the content of HPW gradually decreased. After four cycles, the actual loading decreased from 4.252% to 2.87%. HPW was adsorbed on the support surface in the form of [PW_12_O_40_]^3−^ and interacted with the positive charge on the support surface [9]. The surface potential of SF-HPC was measured by a zeta potentiometer at 16.36 V/cm, indicating that the positive charge of the SF-HPC was low and the interaction force with [PW_12_O_40_]^3−^ was weak, leading to the easy loss of anions, which were consistent with the ICP test and experimental results.

## 4. Conclusions

In summary, the use of the ultra-high specific surface area and macroporous structure of the SF-HPC improved the dispersion of the HPW catalyst, avoiding the agglomeration of HPW and thus reducing the required HPW loading. The optimal HPW/SF-HPC catalyst was successfully prepared with a HPW loading of 5%. The catalytic performance was investigated in the oxidative desulfurization reaction. The optimized reaction conditions were room temperature, 30 min reaction time, O/S = 10, and 0.1 g catalyst per 10 mL of oil. Under the optimal conditions, the sulfur removal rate could reach 100%. The use of SF-HPC reduced the content of the HPW catalyst, which not only reduced the cost of the catalyst but also reduced the pollution caused by phosphorus.

## Figures and Tables

**Table 1:** Structure parameters of the catalysts.

Sample	S_BET_ (m^2^g^−1^)	V_P_ (cm^3^g^−1^)	d (nm)
SF-HPC	3152.46	1.86	2.35
1%HPW/SF-HPC	2805.95	1.77	2.52
5%HPW/SF-HPC	2625.28	1.57	2.40
10%HPW/SF-HPC	2436.84	1.51	2.49
20%HPW/SF-HPC	2080.98	1.27	2.44

**Table 2:** HPW content in the 5%HPW/SF-HPC catalyst.

Run Number	W (wt %)	HPW (wt %)
Fresh	3.257	4.252
1 cycle	3.129	4.085
2 cycles	2.878	3.757
3 cycles	2.616	3.415
4 cycles	2.198	2.870

## Data Availability

Not applicable.

## References

[1] Ogunlaja A.S., Coombes M.J., Torto N., Tshentu Z.R. (2014). The adsorptive extraction of oxidized sulfur-containing compounds from fuels by using molecularly imprinted chitosan materials. React. Funct. Polym..

[2] Yu F.L., Wang Y.Y., Liu C.Y., Xie C.X., Yu S.T. (2014). Oxidative desulfurization of fuels catalyzed by ammonium oxidative-thermoregulated bifunctional ionic liquids. Chem. Eng. J..

[3] Sachdeva T.O., Pant K.K. (2010). Deep desulfurization of diesel via peroxide oxidation using phosphotungstic acid as phase transfer catalyst. Fuel Process. Technol..

[4] Choi A.E.S., Roces S., Dugos N., Wan M.W. (2016). Mixing-assisted oxidative desulfurization of model sulfur compounds using polyoxometalate/H_2_O_2_ catalytic system. Sustain. Environ. Res..

[5] Wu N.J., Li B.S., Ma W., Han C.Y. (2014). Synthesis of lacunary polyoxometalate encapsulated into hexagonal mesoporous silica and their catalytic performance in esterification. Microporous Mesoporous Mater..

[6] Yang P., Zhou S.Y., Du Y., Li J.S., Lei J.H. (2016). Synthesis of ordered meso/macroporous H_3_PW_12_O_40_/SiO_2_ and its catalytic performance in oxidative desulfurization. RSC Adv..

[7] Du Y., Lei J.H., Yang P., Li J.S., Du X.D. (2018). Hierarchical ordered meso/macroporous H_3_PW_12_O_40_/SiO_2_, catalysts with superior oxidative desulfurization activity. J. Porous Mater..

[8] Huang P.C., Liu A.L., Kang L.H., Zhu M.Y., Dai B. (2018). Heteropoly acid supported on sodium dodecyl benzene sulfonate modified layered double hydroxides as catalyst for oxidative desulfurization. New J. Chem..

[9] Pham X.N., Doan H.V. (2018). Activity and stability of amino-functionalized SBA-15 immobilized 12-tungstophosphoric acid in the oxidative desulfurization of a diesel fuel model with H_2_O_2_. Chem. Eng. Commun..

[10] Gao H.C., Wu X.N., Sun D.M., Niu G.L., Guan J.Y., Meng X.F., Liu C.Z., Xia W.D., Song X.J. (2019). Preparation of Core-shell PW_12_@TiO_2_ Microsphere and Oxidative Desulfurization Performance. Dalton Trans..

[11] Zhu Y.F., Zhu M.Y., Kang L.H., Yu F., Dai B. (2015). Phosphotungstic Acid Supported on Mesoporous Graphitic Carbon Nitride as Catalyst for Oxidative Desulfurization of Fuel. Ind. Eng. Chem. Res..

[12] Du Y., Lei J.H., Zhou L.N., Du X.D., Guo Z.R., Li J.S. (2020). Oxidative desulfurization of fuels at room temperature using ordered meso/macroporous H_3_PW_12_O_40_/SiO_2_ catalyst with high specific surface areas. Arab. J. Chem..

[13] Li S.W., Gao R.M., Zhang W., Zhang Y., Zhao J.S. (2018). Heteropolyacids supported on macroporous materials POM@MOF-199@LZSM-5: Highly catalytic performance in oxidative desulfurization of fuel oil with oxygen. Fuel.

[14] Huang P.C., Luo G.Q., Kang L.H., Zhu M.Y., Dai B. (2017). Preparation, characterization and catalytic performance of HPW/aEVM catalyst on oxidative desulfurization. RSC Adv..

[15] Liang Y.R., Wu B.M., Wu D.C., Xu F., Li Z.H., Luo J.W., Zhong H., Fu R.W., Matyjaszewski K. (2011). Ultrahigh surface area hierarchical porous carbons based on natural well-defined macropores in sisal fibers. J. Mater. Chem..

[16] Liu X.W., Liu X.H., Sun B.F., Zhou H.L., Fu A.P., Wang Y.Q., Guo Y.G., Guo P.Z., Li H.L. (2018). Carbon materials with hierarchical porosity: Effect of template removal strategy and study on their electrochemical properties. Carbon.

[17] Zhu Z.G., Ma J.X., Ji C.H., Liu Y., Wang W., Cui F.Y. (2018). Nitrogen doped hierarchically structured porous carbon fibers with an ultrahigh specific surface area for removal of organic dyes. RSC Adv..

[18] Amosa M.K., Jami M.S., Alkhatib M.F.R. (2016). Electrostatic Biosorption of COD, Mn and H_2_S on EFB-Based Activated Carbon Produced through Steam Pyrolysis: An Analysis Based on Surface Chemistry. Equilibria and Kinetics. Waste Biomass Valorization.

[19] Choi A.E.S., Roces S., Dugos N., Arcega A., Wan M.W. (2017). Adsorptive removal of dibenzothiophene sulfone from fuel oil using clay material adsorbents. J. Clean. Prod..

[20] Masoomi M.Y., Bagheri M., Morsali A. (2015). Application of Two Cobalt-Based Metal-Organic Frameworks as Oxidative Desulfurization Catalysts. Inorg. Chem..

[21] Zhang Y., Li G., Kong L.H., Lu H. (2018). Deep oxidative desulfurization catalyzed by Ti-based metal-organic frameworks. Fuel.

[22] Rose M., Korenblit Y., Kockrick E., Borchardt L., Oschatz M., Kaskel S., Yushin G. (2011). Hierarchical Micro-and Mesoporous Carbide-Derived Carbon as a High-Performance Electrode Material in Supercapacitors. Small.

[23] Liang Y.R., Wu D.C., Fu R.W. (2009). Preparation and electrochemical performance of novel ordered mesoporous carbon with an interconnected channel structure. Langmuir.

[24] Wu D.C., Liang Y.R., Yang X.Q., Zou C., Li Z.H., Lv G.F., Zeng X.H., Fu R.W. (2008). Preparation of activated ordered mesoporous carbons with a channel structure. Langmuir.

[25] Li B.S., Liu Z.X., Han C.Y., Ma W., Zhao S.J. (2012). In situ synthesis, characterization, and catalytic performance of tungstophosphoric acid encapsulated into the framework of mesoporous silica pillared clay. J. Colloid. Interf. Sci..

[26] Zhou Y., Chen G.J., Long Z.Y., Wang J. (2014). Recent advances in polyoxometalate-based heterogeneous catalytic materials for liquid-phase organic transformations. RSC Adv..

[27] Wee L.H., Bonino F., Lambert C., Bordiga S., Martens J.A. (2014). Cr-MIL-101encapsulated Keggin phosphotungstic acid as active nanomaterial for catalysing the alcoholysis of styrene oxide. Green Chem..

[28] Luo G.Q., Kang L.H., Zhu M.Y., Dai B. (2014). Highly active phosphotungstic acid immobilized on amino functionalized MCM-41 for the oxidesulfurization of dibenzothiophene. Fuel Process. Technol..

[29] Huang P.C., Liu A.L., Kang L.H., Dai B., Zhu M.Y., Zhang J.L. (2017). Heteropolyacid Supported on Nitrogen-doped Onion-Like Carbon as Catalyst for Oxidative Desulfurization. ChemistrySelect.

[30] Du Y., Yang P., Zhou S.Y., Li J.S., Du X.D., Lei J.H. (2018). Direct synthesis of ordered meso/macrostructured phosphotungstic acid/SiO_2_ by EISA method and its catalytic performance of fuel oil. Mater. Res. Bull..

[31] Zhang Y.Q., Wang R. (2018). Synthesis of Silica@C-dots/Phosphotungstates Core-Shell Microsphere for Effective Oxidative-Adsorptive Desulfurization of Dibenzothiophene with Less Oxidant. Appl. Catal. B Environ..

[32] Yan X.M., Mei Z.K., Mei P., Yang Q.F. (2014). Self-assembled HPW/silica–alumina mesoporous nanocomposite as catalysts for oxidative desulfurization of fuel oil. J. Porous Mater..

[33] Luna M.D.G.D., Wan M.W., Golosinda L., Futalan C., Lu M.C. (2017). Kinetics of Mixing-Assisted Oxidative Desulfurization of Dibenzothiophene in Toluene Using a Phosphotungstic Acid/Hydrogen Peroxide System: Effects of Operating Conditions. Energ. Fuel.

[34] Han W., Rui W., Zhang Y.Q., Dou S.Y., Olga S., Vladimir K. (2018). Oxidative Removal of Organo-sulfur Species via H_2_O_2_ Oxidation Catalyzed by Lacunary Keggin 11-tungstophosphates. Catal. Lett..

